# Impact of burnout, secondary traumatic stress and compassion satisfaction on hand hygiene of healthcare workers during the COVID‐19 pandemic

**DOI:** 10.1002/nop2.786

**Published:** 2021-02-19

**Authors:** Qian Zhou, Xiaoquan Lai, Zhaoyang Wan, Xinping Zhang, Li Tan

**Affiliations:** ^1^ School of Medicine and Health Management Tongji Medical School Huazhong University of Science and Technology Wuhan China; ^2^ Tongji Hospital Tongji Medical College Huazhong University of Science and Technology Wuhan China; ^3^ Department of Critical Care Medicine Peking Union Medical College Hospital Chinese Academy of Medical Sciences and Peking Union Medical College Dongcheng China

**Keywords:** burnout, compassion satisfaction, hand hygiene, medical aid, secondary traumatic stress

## Abstract

**Aim:**

To assess the prevalence of burnout, secondary traumatic stress, and compassion satisfaction and explore their impacts on self‐reported hand hygiene among medical aid teams in the COVID‐19 period in Wuhan, China.

**Design:**

Cross‐sectional study.

**Method:**

A total of 1,734 healthcare workers from 17 medical aid teams were surveyed. The survey included burnout, secondary traumatic stress and compassion satisfaction measured by the professional quality of life scale and self‐reported hand hygiene. Data were collected between 5–7 March 2020. Multiple regression analyses were performed.

**Results:**

Burnout and secondary trauma stress were at low and average levels, and compassion satisfaction was at average and high levels. Burnout was negatively associated with hand hygiene, while compassion satisfaction was positively associated. Hospital administrators should pay attention to burnout and compassion satisfaction to improve infection control behaviours. Management of healthcare workers in our study may be constructive in emerging infectious diseases.

## 1 INTRODUCTION

COVID‐19 is an emerging infectious disease caused by SARS‐CoV‐2. With this virus spread globally, WHO declared the outbreak a Public Health Emergency of International Concern on 30th January, and a pandemic on 11th March. Up to the 8th of July, 11.7 million people were diagnosed with COVID‐19 across the world, which contained a large number of healthcare workers (HCWs). This pandemic of COVID‐19 underlined how crucial it is to develop a comprehensive system to sustain the health of HCWs at health facilities (Shah et al., 2020; Godlee, 2020).

Medical aid teams were sent to assist Hubei province to confront the problem of manpower shortage in the initial stage of COVID‐19 in Hubei province. On 1 March 2020, a total of 344 medical aid teams with a total of 42,322 HCWs were dispatched across China. Most of them were nurses, with the number of 28,679. Nurses are irreplaceable in medical aid teams because they are the main force and can provide professional care for critical patients. The treatment for critical COVID‐19‐infected patients needs quite a lot of nursing work, including airway management, ECMO nursing, ventilation in the prone position and bedside hemofiltration. HCWs had a high risk of experiencing traumatic symptoms when confronted with emerging infectious diseases (i.e. SARS and SARS‐CoV‐2), including burnout, secondary traumatic stress, anxiety and depression (Chew, Lee, et al., 2020; Chew, Ngiam, et al., 2020; Sim & Chua, 2004; Tan et al., 2020). That is because they suffered more bio‐psycho‐socio‐behavioural risk factors than the general period, including adapting to a different culture, the disruption of family and social networks, the isolated living environment and long working hours, etc (Li et al., 2018).

Infection control behaviours, including hand hygiene behaviours, are crucial in the COVID‐19 pandemic, and the high compliance of hand hygiene is the cornerstone to protect HCWs and keep patients safe. The inappropriate compliance of hand hygiene may induce terrible cross‐infection among HCWs and patients, causing unnecessary morbidity, mortality and healthcare cost (Erasmus et al., 2010). Current studies have explored the impacts of knowledge, attitude, awareness and socio‐demographic characteristics on hand hygiene comprehensively (Akinyinka et al., 2019; Colindres et al., 2018; Luangasanatip et al., 2015; Raab et al., 2020). For example, Hosseinialhashemi et al. (2015) found that knowledge and attitude have a positive correlation with hand hygiene practice. However, the impact of psychological status is little known. Although Colindres et al. (2018) and Manomenidis et al. (2019) found that burnout is the predictor of lower hand hygiene, the small sample size limited the generalization of the two studies. In summary, the evidence concerning the influence of psychological status on hand hygiene was limited.

Burnout, secondary traumatic stress and compassion satisfaction constituted professional quality of life (ProQOL), which is defined as “the quality one feels about their work as a helper” (Shen et al., 2015; Stamm, 2010; Wang, Chudzicka‐Czupała, et al., 2020; Wang, Okoli, et al., 2020). ProQOL is appropriate for individuals exposed to potentially traumatizing events, as a result of paid or volunteer work, like medical aid teams (Stamm, 2010). As the positive aspect of ProQOL, compassion satisfaction refers to positive feelings or a sense of self‐efficacy derived from helping others (Christian‐Brandt et al., 2020). As the negative aspects of ProQOL, burnout is related to frustration with work situations and colleagues, whereas secondary traumatic stress is unique to the healthcare profession and patient care (Polat et al., 2020). Researchers found that negative aspects of ProQoL in HCWs are associated with poor quality of care, including poor quality and safety measures and unprofessional behaviour, etc. (Harolds, 2019; Tawfik et al., 2019). The positive aspect, compassion satisfaction, means that individuals feel happy with the work and want to continue, and worse compassion satisfaction was reported to reduce the standards of care (Dasan et al., 2015; Stamm, 2010).

Based on the existing studies, we found that psychological statuses were crucial influencing factors of HCWs' behaviours. Studies on the association between psychological status and hand hygiene behaviours can provide the premise for the intervention aiming to improve the compliance of hand hygiene. However, current evidence in the prevalence of HCWs' ProQoL and its impact on hand hygiene behaviours or even infection prevention and control (IPC) measures were limited. The gap remains in the impacts of psychological status on the hand hygiene behaviours, especially in the emerging infectious disease period. Thus, this study aimed to assess the prevalence of burnout, secondary traumatic stress and compassion satisfaction among HCWs in medical aid teams and explore their impacts on hand hygiene in the context of the COVID‐19 pandemic. The following research questions were addressed: what level are burnout, secondary traumatic stress, and compassion satisfaction, and to what extent they influenced hand hygiene in HCWs in medical aid teams. We hypothesized that.

1. HCWs in medical aid teams experience a high level of burnout and secondary traumatic stress and a low level of compassion satisfaction.
2. Burnout and secondary traumatic stress are negatively associated with hand hygiene.
3. Compassion satisfaction is positively associated with hand hygiene.

## 2 METHODS

### 2.1 Study design and settings

A cross‐sectional study was conducted in the Optics Valley Branch of Tongji Hospital in Huazhong University of Science and Technology, Wuhan, China. A total of 828 beds were available to treat COVID‐19 patients, and 1,462 patients were admitted between 2 February–30 March 2020. HCWs were mainly composed of 17 medical aid teams from other hospitals across the whole country.

The hospital adopted a series of measures to manage the medical aid teams including infection control education and psychological support. The management detail can be seen in Table 1. In the period of medical aid, no HCWs from medical aid teams were infected with COVID‐19.

**TABLE 1:** Measures and details in the management of medical aid teams

Measures	Details
Work scheduling	Nurses worked 4 hours every day; doctors worked 6 hours every day. Days off were set based on the number of patients. One nurse cared for no more than two patients, based on the severity of patients.
Protective equipment	A protective suit, a gown, a goggle or a face shield, three layers of gloves, a pair of ordinary shoe cover and a pair of waterproof long shoe cover were provided every shift. Adult diapers were used in work time.
Education and meeting	Educations including diagnosis, treatment and infection control measures were developed by leader groups, and delivered to doctors and nurses by department managers via video or text. Meetings were held among the managers every week, discussing the flow, position arrangement, obligation and measure improvement, and delivered to doctors and nurses by department managers via text. HCWs must wear masks and keep one‐metre distance with each other in the meetings.
Health measures	Daily surveillance of the health status and the risk exposure of HCWs were conducted. Nasopharyngeal swabs and blood samples were collected every month. The flow of treatment for HCWs was formulated, if HCWs were suspected or diagnosed, etc.
Psychological support	Psychological supports were provided by psychological professors via telephone. The telephone numbers were given to HCWs; they can contact if necessary.
Financial support	Salaries of HCWs in medical aid teams included one part from the original hospital and the other from local subsidies of Wuhan from district service centre, aid hospital and the Red Cross.
Accommodation and transportation measures	HCWs of medical aid teams resided in hotel, which also provided meals. HCWs transported between the hotel and the hospital via special shuttle bus.

### 2.2 Data collection

A structured self‐administered questionnaire was used to collect data from 5–7 March 2020. HCWs in medical aid teams who were willing to take part in the study submitted the finished online questionnaires, which included self‐reported hand hygiene behaviours, the ProQOL scale covering burnout, secondary traumatic stress and compassion satisfaction, and socio‐demographic characteristics. A total of 1,734 HCWs were surveyed.

### 2.3 Measurements

#### 2.3.1 Dependent variables

##### Self‐reported hand hygiene behaviours

Outcome indicators were self‐reported hand hygiene behaviours because the compliance of the other important measures was nearly 100% or hard to measure. For example, the compliance of the use of personal protective equipment (PPE) was approximately 100% due to the strict management of medical aid teams. Hand hygiene behaviours were measured by 18 moments of hand hygiene based on the IPC guideline issued by the National Health Commission of the People's Republic of China (2020), along a five‐point Likert scale that ranges from 1 (never)–5 scores (very often), reflecting the frequency of hand hygiene. 18 moments consisted of moments before touching a patient, before the aseptic procedure, after body fluid exposure risk, after touching a patient, after touching patient surroundings, during work on a soiled body site to a clean body site on the same patient, before putting on PPE, before after and during removing all the PPE, before wearing a glove, after removing the glove, arriving at and leaving the ward, before drinking, before and after using the toilet, before returning to the place of residence(Zhou et al., 2020).

#### 2.3.2 Independent variables

##### ProQOL

The ProQOL Scale of Chinese version was adopted to assess burnout, secondary traumatic stress and compassion satisfaction using a five‐point Likert scale that ranges from 1 (never)–5 (very often) (Shen et al., 2015). In this study, internal consistency ranged from acceptable to strong: burnout (*α* = 0.869; good), secondary traumatic Stress (*α* = 0.756; acceptable) and compassion satisfaction (*α* = 0.936; strong). And construct validity was shown to be strong in the previous study (Wang, Chudzicka‐Czupała, et al., 2020; Wang, Okoli, et al., 2020). The 30‐item ProQOL scale consists of three subscales: compassion satisfaction (10 items), burnout (10 items) and secondary traumatic stress (10 items). Items 1, 4, 15, 17 and 29 are reverse scored. Each subscale score of 22 or less suggests low levels of compassion satisfaction, burnout and secondary traumatic stress; scores of 23–41 indicate average levels; and 42 and above suggest high levels (Stamm, 2010).

##### Socio‐demographic characteristics

The socio‐demographic characteristics variables were collected, including gender (male versus female), career type (doctor versus nurse), age, work year and workload. The workload was measured using a self‐reported five‐point Likert scale (ranged from very low to very high).

### 2.4 Statistical analysis

Categorical variables were described using percentages and frequency rates. Continuous variables were described using means and standard deviations (*SD*). T tests and one‐way analyses of variance were used to compare self‐reported hand hygiene behaviours reported in different levels of burnout, secondary traumatic stress and compassion satisfaction score. Multiple regression analyses were performed to determine the impact of burnout, secondary traumatic stress and compassion satisfaction on self‐reported hand hygiene behaviours adjusted the socio‐demographic characteristics. All statistical analyses were performed using IBM SPSS Version 20.0 (IBM, New York, NY, USA).

### 2.5 Ethics

This study was approved by the Ethics Committee of Tongji Medical College, Huazhong University of Science and Technology (IORG: IORG0003571). All participants were enrolled in the investigation using the principles of informed consent and confidentiality.

## 3 RESULTS

### 3.1 Socio‐demographic characteristics

A total of 1,383 (79.76%) nurses and 351 (20.24%) doctors were surveyed, including 1,305 (75.26%) female and 429 (24.74%) male. The mean and *SD* of age and work year were 33.33 ± 6.39 and 10.90 ± 6.48, respectively. Most HCWs reported neutral (936[53.98%]) and high (661[38.12%]) workload.

### 3.2 Prevalence of burnout, secondary traumatic stress and compassion satisfaction

None of the HCWs reported high burnout, with 69.61% at the low level and 30.39% at the average level. Most HCWs reported the low (33.33%) and average (66.21%) level in secondary traumatic stress, and the average (49.65%) and high (49.71%) level in compassion satisfaction. (Table 2).

**TABLE 2:** ProQOL means, prevalence and *SD*

ProQOL	Mean (min–max)	Prevalence (*n* [%])		*SD*
Burnout	19.42 (10.00–40.00)	Low (≤22)	1,207 (69.61)	5.73
		Average (23–41)	527 (30.39)	
Secondary traumatic stress	24.76 (10.00–49.00)	Low (≤22)	578 (33.33)	5.09
		Average (23–41)	1,148 (66.21)	
		High (≥42)	8 (0.46)	
Compassion satisfaction	41.43 (10.00–50.00)	Low (≤22)	11 (0.63)	6.49
		Average (23–41)	861 (49.65)	
		High (≥42)	862 (49.71)	

### 3.3 Relationship between hand hygiene and burnout, secondary traumatic stress and compassion satisfaction

According to univariate analyses, the lower burnout and higher compassion satisfaction in HCWs were associated with higher self‐reported hand hygiene behaviours in hand hygiene (*p* < .001). HCWs with different secondary traumatic stress and compassion satisfaction reported different levels of hand hygiene. (Table 3).

**TABLE 3:** Univariate analysis of self‐reported hand hygiene behaviours and ProQOL

	Mean (*SD*)	*p*
Burnout		
Low	4.86 (0.22)	<.001
Average	4.70 (0.42)	
Secondary traumatic stress		
Low	4.86 (0.29)	<.001
Average	4.79 (0.31)	
High	4.96 (0.07)	
Compassion satisfaction		
Low	4.16 (1.28)	<.001
Average	4.74 (0.34)	
High	4.89 (0.19)	

According to multivariate analyses, burnout was negatively associated with hand hygiene (Coef. = −.088, *p* < .001). HCWs with higher compassion satisfaction reported higher hand hygiene behaviours: HCWs with a high level of compassion satisfaction reported better hand hygiene compared to the average level (Coef. = .661 *p* < .001), and HCWs with the average level of compassion satisfaction reported better hand hygiene compared to the low level (Coef. = .556, *p* < .001). Besides, HCWs with older age were negatively associated with self‐reported hand hygiene behaviours (Coef. = −.005, *p* = .079). (Table 4).

**TABLE 4:** Multivariable regression analysis of ProQOL with self‐reported hand hygiene behaviours

	Coef.	Std. Err.	*p*
Burnout	−.088	0.019	<.001
Compassion Satisfaction			
Low (ref)			
Average	.556	0.089	<.001
High	.661	0.090	<.001
Age	−.005	0.003	.079

## 4 DISCUSSION

This study assessed the prevalence of burnout, secondary traumatic stress and compassion satisfaction based on the ProQOL scale and explored their impacts on self‐reported hand hygiene behaviours among medical aid teams during the COVID‐19 pandemic. Most HCWs experienced a low and average level of burnout and secondary traumatic stress and average and high level of compassion satisfaction. HCWs with lower burnout and higher compassion satisfaction reported higher compliance with hand hygiene.

Self‐reported hand hygiene behaviours in our study were consistent with previous studies during the MERS‐CoV outbreak (Alshammari et al., 2018). HCWs reported higher compliance of hand hygiene concerning those behaviours that perceived as self‐protective and having a high risk of cross‐contamination or cross‐infection, such as after body fluid exposure risk, after removing a glove and PPE (Lambe et al., 2019; WHO & WHO Patient Safety, 2009). Interestingly, we can find that the level of burnout and secondary traumatic stress is lower and compassion satisfaction is higher than previous studies conducted in China and other countries, not in the period of emerging infectious diseases (Dasan et al., 2015; Shen et al., 2015; Wang, Chudzicka‐Czupała, et al., 2020; Wang, Okoli, et al., 2020; Woo et al., 2020). In general, HCWs reported more psychological burden, when they were exposed to the centre of the outbreak, had experience in caring for the infected or suspected patient and in the frontline (Kim & Choi, 2016; Lai et al., 2020). The lower level of the negative and higher level of positive ProQOL we obtained may be associated with the effect of activities acting on HCWs to confront the pandemic. The activities included careful management from the hospital, the positive report, and higher social status from the public during the pandemic. The zero infection of HCWs in medical aid teams also proves the effective management in medical aid teams It is also observed that the environment of management and public may be highly associated with the burnout and compassion satisfaction of HCWs (Chung et al., 2020; Saeidi et al., 2020).

As for the impact to hand hygiene behaviours, we find that burnout, compassion satisfaction and age are influencing factors of hand hygiene behaviours. More precisely, lower burnout, higher compassion satisfaction and younger age promote hand hygiene behaviours, which is consistent with previous studies. High burnout in HCWs is associated with poor‐quality care, including poor adherence to management guidelines, medical error, healthcare‐associated infections and hand hygiene (Manomenidis et al., 2019; Tawfik et al., 2019). And Dasan et al. (2015) found that consultants with lower compassion satisfaction reduce their standards of care because they are more irritable with patients or colleagues. The reasons why burnout is associated with infection control mainly include three assumptions. First, HCWs with higher burnout may be less likely to follow the standard behaviour or notice errors and omissions, which contribute to the reduction of compliance with infection control (Cimiotti et al., 2012). Second, the emotional and cognitive detachment and the development of cynicism associated with burnout may cause inadequate infection control behaviours and personal inefficiency (Galletta et al., 2016). Third, bad infection control behaviours can increase burnout in HCWs in turn, by increasing the nosocomial infection incidence, the length of stay and higher workload in HCWs (Tawfik et al., 2017).

Similar to previous studies, younger age can be associated with a higher score on hand hygiene (Erasmus et al., 2010). Oh also found that the infection control experience is an influencing factor of hand hygiene behaviours (Oh, 2018). It is worth mentioning that recent studies have also found that low IPC behaviour (wearing masks) could be the contributing cause of negative psychological factors (Wang, Chudzicka‐Czupała, et al., 2020; Wang, Okoli, et al., 2020). Based on this, future studies can further investigate the causal relationship between psychological factors and IPC behaviours, including hand hygiene, wearing masks and use of protective equipment.

Relevant intervention is the approach to reduce burnout and increase compassion satisfaction. The individual‐based interventions include mindfulness‐based technique, educational, emotional empowerment (Janssen et al., 2018; West et al., 2018). The organization‐directed interventions include increased support for clinical work, structural changes, routine staff meetings and improving the work environment (Bresesti et al., 2020). To improve hand hygiene behaviours and compassion satisfaction, and relieve burnout in a pandemic, the managers should take more into consideration and intervention targeting should be performed. As we found that relatively lower burnout and compassion satisfaction in medical aid teams, the management of HCWs adopted in our study (Table 1) may be constructive for future medical aid teams.

This work reveals implications and experiences for emergent preparedness and HCW resource management. Firstly, experiences of management of HCWs in medical aid teams in our study have referential meaning to combat COVID‐19, for the achievement of zero infection, lower burnout and higher compassion satisfaction in these populations. Secondly, as lower burnout and higher compassion satisfaction are associated with better hand hygiene behaviours, the improvement of burnout and compassion should be emphasized, which can be obtained by targeting intervention. Thirdly, the public and managers should respect HCWs and emphasize their importance, which may be highly associated with burnout in HCWs.

### 4.1 Limitations

The limitation of this study is that self‐reported hand hygiene behaviours may be overestimated compared to observed hand hygiene behaviours, because HCWs may respond to questions in a way that they believe is socially acceptable rather than the fact, namely “Social desirability” (Edwards, 1958). Second, we did not assess the effect of the intervention of the management of medical aid teams, because the baseline data were unavailable.

## 5 CONCLUSIONS

Most HCWs in medical aid teams experience lower level burnout, and a higher level of compassion satisfaction during the COVID‐19 pandemic compared to the general period. Lower burnout, higher compassion satisfaction and younger age are associated with higher self‐reported hand hygiene behaviours. Burnout and compassion satisfaction in HCWs should be emphasized and need interventions targeting the management of burnout and compassion satisfaction. The management of HCWs in our study may be constructive for future medical aid teams.

## CONFLICT OF INTEREST

The authors declare that they have no competing interests.

## AUTHOR CONTRIBUTIONS

QZ: Conceptualization, data analysis, data interpretation, and manuscript draft. XPZ: Conceptualization and manuscript draft. LT and XQL: Data collection. ZYW: Process of drafting.

## Data Availability

The data that support the findings of this study are available on request from the corresponding author. The data are not publicly available due to privacy or ethical restrictions.
